# Chemotaxis of *Dictyostelium discoideum:* Collective Oscillation of Cellular Contacts

**DOI:** 10.1371/journal.pone.0054172

**Published:** 2013-01-17

**Authors:** Edith Schäfer, Marco Tarantola, Elena Polo, Christian Westendorf, Noriko Oikawa, Eberhard Bodenschatz, Burkhard Geil, Andreas Janshoff

**Affiliations:** 1 Institute of Physical Chemistry, Georg-August-University Göttingen, Göttingen, Germany; 2 Laboratory for Fluid Dynamics, Pattern Formation and Biocomplexity (LFPB), Max Planck Institute for Dynamics and Self-Organization, Göttingen, Germany; 3 Institute of Nonlinear Dynamics, Georg-August-University Göttingen, Göttingen, Germany; Université de Genève, Switzerland

## Abstract

Chemotactic responses of *Dictyostelium discoideum* cells to periodic self-generated signals of extracellular cAMP comprise a large number of intricate morphological changes on different length scales. Here, we scrutinized chemotaxis of single *Dictyostelium discoideum* cells under conditions of starvation using a variety of optical, electrical and acoustic methods. Amebas were seeded on gold electrodes displaying impedance oscillations that were simultaneously analyzed by optical video microscopy to relate synchronous changes in cell density, morphology, and distance from the surface to the transient impedance signal. We found that starved amebas periodically reduce their overall distance from the surface producing a larger impedance and higher total fluorescence intensity in total internal reflection fluorescence microscopy. Therefore, we propose that the dominant sources of the observed impedance oscillations observed on electric cell-substrate impedance sensing electrodes are periodic changes of the overall cell-substrate distance of a cell. These synchronous changes of the cell-electrode distance were also observed in the oscillating signal of acoustic resonators covered with amebas. We also found that periodic cell-cell aggregation into transient clusters correlates with changes in the cell-substrate distance and might also contribute to the impedance signal. It turned out that cell-cell contacts as well as cell-substrate contacts form synchronously during chemotaxis of *Dictyostelium discoideum* cells.

## Introduction

*Dictyostelium discoideum* (*D. discoideum*) is a life form that proliferates as single amebas and when starved, aggregates into a mound of approximately 10^5^ cells that eventually turns into a migrating slug, which further differentiates into a fruiting body to facilitate spore dispersal. The formation of multicellular structures is governed by chemotactic responses to periodic self-generated signals of extracellular cyclic adenosine 3′,5′- monophosphate (cAMP) [1], [2], [3], [4]. The transition from the single ameba state to a multicellular entity is characterized by a spontaneous change from a quiescence state to periodic synthesis and release of extracellular cAMP encoding cell-density information in the frequency of the oscillations and providing a directional signal for migration [3], [5], [6].

Cells produce cAMP when they encounter an increase in the extracellular cAMP concentration. In turn, the cAMP molecules are decomposed by the enzyme phospodiesterase that is expressed by the cell continuously. This way, pulses of cAMP can be sustained [2], [7], [8], [9]. During the spatiotemporal evolution, wave emitting centers are generated that later act as aggregation centers [10], [11]. The immediate response of cells to cAMP addition leads to cringing [12], [13] followed by spreading and pseudopodium extension into the direction of the stimulus [5], [14], [15]. The cells resume their prestimulus morphology roughly after 2–3 minutes [13], [16], [17], [18]. Individual cells, which are able to detect a discrepancy of as much as 5 occupied receptors between front and back of the cell body, migrate into the direction of increasing cAMP concentration producing spatiotemporal waves that can be visualized by dark-field microscopy [19], [20]. These variations in light scattering properties are believed to originate from cell shape or cell density changes that occur during the chemotactic cycle producing time periods of 5–6 minutes [10], [21]. Resting and therefore predominately round cells scatter less light than moving ones that display an elongated shape. Hence, cell shape changes give rise to the observed pattern in dark field microscopy [16], [19], [22]. So far, however, the collective shape oscillations of amebas in the context of small ensembles have only been covered by few authors [23], [24], [25], [26]. Although it is well know that the cell populations show wave propagation, the heterogeneity at small scale has been sparsely addressed [19]. Propagating waves of extracellular cAMP act also as a mediator of cell-cell signaling to coordinate movements of large amounts of amebas. However, cell-substrate contacts of small ensembles in 2-D during chemotaxis have yet to be studied.

So far, it is unclear, if the initiation of the periodic behavior relies on the synchronization of cells that autonomously oscillate or whether individual cells remain quiescent unless the entire population starts to oscillate [11], [27]. Recently, Gregor et al. showed that the initiation of oscillations is highly dynamic and collective, since extracellular threshold concentration of cAMP and the oscillation frequency of the response cannot be attributed solely to those at the single cell level [11]. Gregor et al. believe that extracellular cAMP accumulates so that, at the peak of the synchronized pulses, cells become transiently oscillatory. The combination of global and local strategies allows cells to determine the overall maximum of extracellular cAMP concentration and then to aggregate as the pulses become self-sustainable [11], [27].

Cell migration has been extensively studied using cell shape as readout with descriptors such as cell length or the velocity of the cell centroid [23]. Principal component analysis has been frequently applied to obtain a reduced set of dominant components of cell shape changes during migration [9], [28]. However, it remains to be elucidated what kind of morphological changes are associated with cAMP oscillations both on the single cell level as well as on the level of small cell ensembles, in which cell-cell contacts are important for the overall organization of the aggregation [29], [30], [31], [32].

Recently, we reported on impedance oscillations generated by small ensembles of amebas using electric cell-substrate impedance sensing (ECIS) [33]. We could show that ensembles of *D. discoideum* on gold electrodes induce periodic impedance changes. However, the periodic changes of the impedance signal could not be unequivocally assigned to particular changes in cell shape simultaneously monitored by optical microscopy on the electrodes. Hence, the question what kind of changes in cellular organization on the level of single amebas as well as small ensembles are responsible for the observed periodic impedance spikes remains unanswered.

A number of potential contributions to the overall impedance of the electrode might be envisioned. Possible causes of impedance spikes comprise the periodic changes in i) the number of amebas on the electrode surface, ii) changes of the electrode area occupied by cells, iii) shape changes, periodic variations in iv) cell-cell distance or in v) cell-substrate distance.

**Figure 1:**
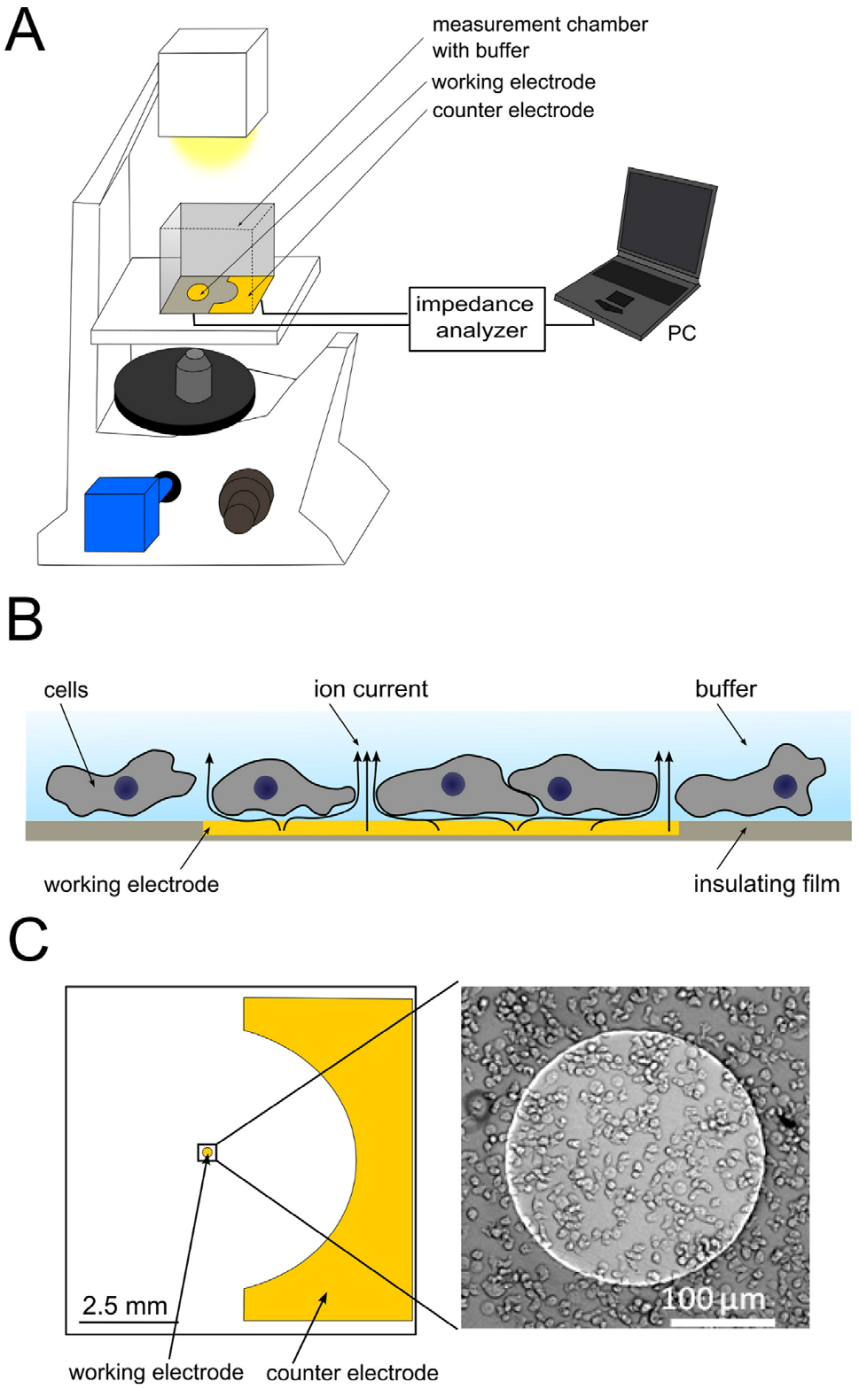
Scheme of experimental setup. A) Scheme illustrating the experimental setup. It comprises an electric cell-substrate impedance sensing (ECIS) setup on top of an inverted microscope. The complex impedance of the small counter electrode is measured with an impedance analyzer (SI 1260). B) Sketch of a *D. discoideum* covered gold-film electrode. Most of the current flows through the buffer channels under and between the cells at the utilized measuring frequency (4 kHz). C) Scheme of an ECIS measurement chamber comprising a circular working electrode (area: 5×10^−4^ cm^2^) and a counter electrode (area: 0.15 cm^2^) together with an optical micrograph of *D. discoideum* amebas in glucose –free buffer on the working electrode.

Here, we report on direct and indirect spatiotemporal correlation of video microscopy (bright field and total internal reflection fluorescence (TIRF) microscopy) with time-resolved impedance recordings (Fig. 1). In this work we will demonstrate that the main contribution to the impedance oscillations are temporal changes of the cell-substrate distance, i.e. a smaller cell-substrate distance causes an increase in impedance. These findings were successfully corroborated with acoustic resonator measurements using a quartz crystal microbalance with dissipation monitoring (D-QCM) that display also variations in cell-substrate distance as inferred from the correlation between the oscillating dissipation signal and period changes of the resonance frequency shifts of the quartz resonator. Besides, periodic changes in the local cell-cell distance reproduce both signal shape and oscillation frequency of the impedance signal. We show that this regular formation of small cell clusters is partly responsible for impedance shifts, in which larger clusters produce higher impedance signals at otherwise constant overall electrode coverage with amebas.

## Results

**Figure 2:**
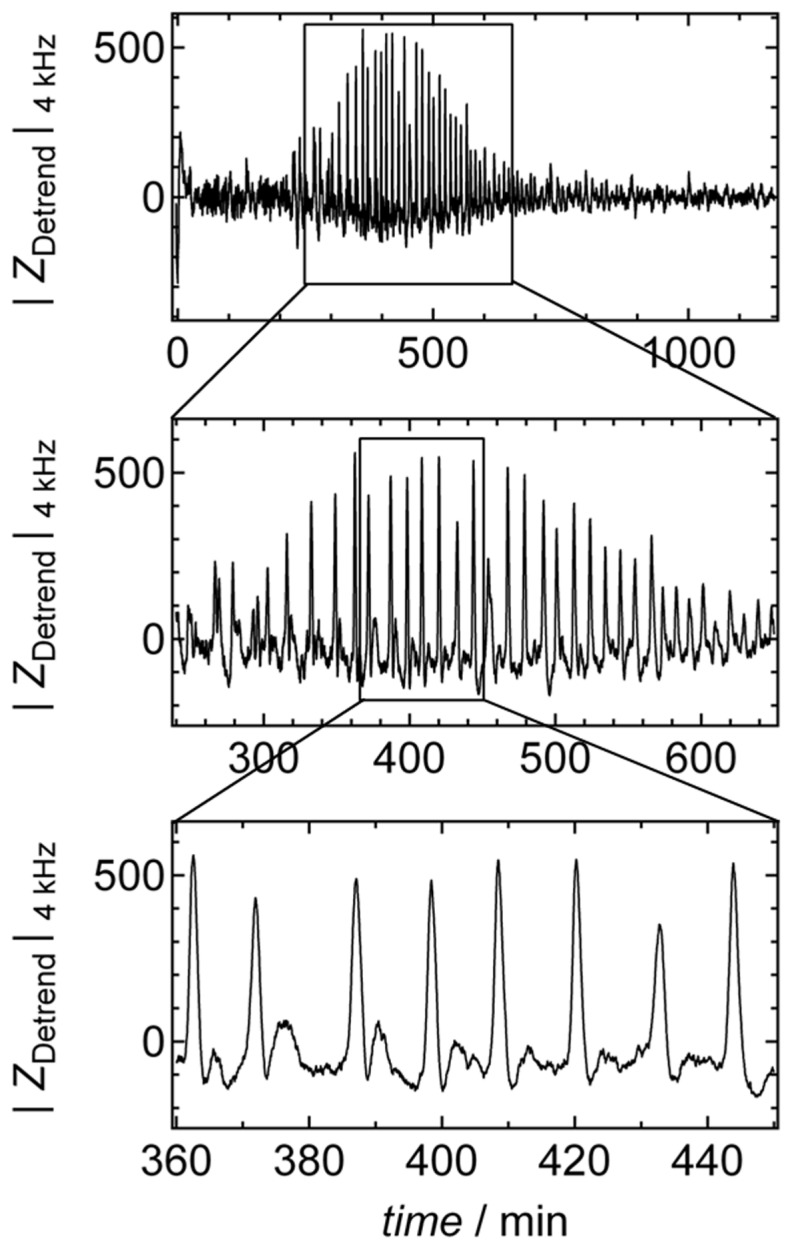
Impedance signal of *D. discoideum*. Magnitude of detrended impedance signal at 4 kHz |*Z*_Detrend_|_4 kHz_ of *D. discoideum* (3,750 cells mm^−2^ added in Sorenseńs buffer) as shown in Figure 1 C. Cells were seeded at *t* = 0 min on a circular gold electrode (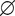 = 250 µm). Boxes highlight magnification of the impedance signal. Data were smoothed by subtracting a moving average algorithm (box size: 800 points) to remove long-term trends.

The primary goal of this study was to elucidate the origin of impedance oscillations generated by starved *D. discoideum* amebas that have been cultured on small gold electrodes. During chemotaxis, *D. discoideum* amebas seeded on micrometer-sized gold electrodes show strong impedance oscillations with amplitudes well beyond 10% of the overall signal (Fig. 2, Fig. S1, Supporting Information). In a previous publication, we speculated that these impedance oscillations might be attributed to synchronous shape changes of amebas similar to what is inferred from optical density oscillations [33]. However, in our previous publication, we could not find clear evidence for this claim since synchronously recorded optical micrographs did not reveal periodic shape changes, which would explain the observed impedance oscillations.

In order to identify correlations between optical micrographs and impedance oscillations, video microscopy was carried out simultaneously with impedance measurements recorded at a single generator frequency. It turned out that a generator frequency of 4 kHz provides the highest signal to noise ratio for impedance fluctuations produced by migrating amebas. The general setup comprising a lock-in amplifier to monitor amplitude and phase shift of voltages applied across a shunt resistor (1 MOhm) is shown in Figure 1. The measured impedance changes solely originate from impedance changes produced by cells adhering onto the small working electrode. The size difference between the working electrode and the substantially larger counter electrode ensures that only the working electrode contributes to the overall impedance (see Experimental Methods). Notably, the working electrode is smaller than the thickness of an individual spiral arm (500–1000 nm) typically observed for starving *D. discoideum* amebas (Fig. 1C) [2]. Even the size of the sample chamber is smaller than the typical extension of *Dictyostelium discoideum* aggregation clusters. The cells on top of the electrode were simultaneously imaged with bright field microscopy using an inverted microscope. Figure 2 shows the outcome of the key experiment, in which *D. discoideum* amebas (3750 cells mm^−2^) were seeded in glucose-free buffer on a circular gold electrode producing periodic impedance fluctuations 3–5 h after starving of the cells.

The time period was found to be in the range of 6–12 min in good accordance with data from optical density measurements [22], [34]. This oscillation, which is a consequence of cAMP-based chemotaxis, usually persists for several hours. During this time the overall impedance frequently decreases (Fig. S1, Supporting Information). The time period between two impedance spikes decreases after an initial phase from (10–12 min) down to 6–7 min.

We addressed the question on the origin of the impedance oscillations during chemotaxis by measuring a number of descriptors derived from video microscopy such as *ameba number density*, *circularity* of the cells, *number of cell-cell contacts* and *occupied area* of the electrode with amebas (Fig. S2, Supporting Information). Therefore, bright field images were acquired every 10 seconds, while the impedance signal was continuously monitored with a time resolution of 1 second. For cell-substrate analysis, it was, however, not possible to carry out TIRF microscopy through the gold electrodes due to quenching of fluorescence emitted by dyes located close to the gold surface [35], [36]. Hence, the strategy was to first determine a correlation between impedance signals and bright field microscopy on the one hand and to find a correlation between TIRF microscopy and bright field imaging on the other hand. This allows eventually identifying a relationship between impedance time series and time-elapsed TIRF images (*vide infra*). Since the cell substrate distance is a major source of cellular impedance in other systems on planar electrodes like epithelial and endothelial cells causality can also be expected in the case of starving amebas.

### Fluctuation in Number Density and Area Occupation

Generally, the impedance signals produced by ECIS measurements depend on the surface coverage of the working electrode [37] rendering the number of amebas a potential candidate for the observed impedance oscillation during chemotaxis of *D. discoideum*. Fluctuations in number density of cells should be mirrored in the impedance signal leading to an increase in impedance if the number of amebas increases and vice versa. Even if the number density of amebas on the electrode does not represent or follow chemotactically driven oscillation, it will at least contribute to the noise level of the impedance signal. Besides, the overall covered area, which is nothing else than the number of amebas *N,* times the mean area of amebas *A* on the electrode, changes the impedance signal. Wegener and coworkers showed that the coverage of the electrode with cells contributes significantly and at larger frequency in a linear fashion to the impedance signal. For instance, the capacitance recorded at 40 kHz decreases linearly with decreasing coverage since the overall membrane area dominates impedance in this frequency regime [37].

**Figure 3:**
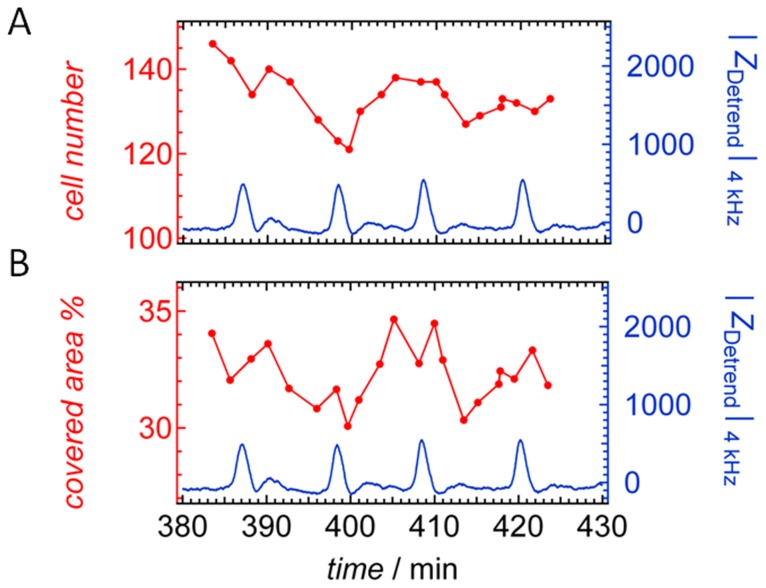
Cell number and covered area of the electrode. A) Temporal evolution of the number of *D. discoideum* cells on the electrode. B) Ameba-covered area of the ECIS-electrode obtained from bright field microscopy (red). Simultaneously recorded impedance |*Z*_Detrend_|_4 kHz_ (blue).

Figure 3 shows time series of cell number and covered area in % from bright field video microscopy of an electrode together with the corresponding impedance changes that have been recorded simultaneously. Cell number on the electrode was determined manually as well as by a fully automated segmentation analysis (see Supporting Information for longer time traces, Fig. S2). Generally, manual analysis was less erroneous due to limited capability of the software to recognize cell borders. The number of *D. discoideum* on the gold coated electrode fluctuates between 120 and 140 amebas and these fluctuations, Δ*N,* displays only a poor correlation with the oscillating impedance signal Δ|*Z*| (Fig. 3 A, Fig. S2). Data analysis carried out over a larger time period (Fig. S2, Supporting Information) revealed a cross-correlation coefficient (eq. 1) of 0.176 beteween Δ*N* and impedance.
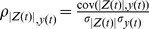
(1)


|*Z*(*t*)| is the time series of the magnitude of complex impedance recorded at 4 kHz, *y*(*t*) the corresponding observable that has been recorded simultaneously (in this case cell number), σ_|*Z*(*t*)|_ the variance of |*Z*(*t*)|, σ_|*y*(*t*)|_ the variance of *y*(*t*) and cov(|*Z*(*t*)|,*y*(*t*)) the covariance. *ρ*_|*Z*(*t*)|,y(*t*)_ might assume values in between –1 and 1 corresponding to a perfect decreasing linear relationship and a perfect positive increasing linear relationship, respectively. Table 1 summarizes the various cross-correlation coefficients between impedance time traces simultaneously recorded with other parameters such as fluctuations in cell number Δ*N*, covered area with of amebas Δ*A*, and circularity Δ*C* (*vide infra*). In order to determine the occupied surface area, cells were segmented manually and the mean area per cell computed (Fig. 3 B). Longer time traces of the mean area of amebas evaluated with cell segmentation software are shown in Fig. S2, Supporting Information. Cross-correlation between corresponding impedance time traces and fluctuations in the mean area per cell Δ*A* is slightly smaller (*ρ*_|*Z*(*t*)|,*A*(*t*)_ = 0.138) than the cross-correlation between number density and impedance data (table 1). Notably, all traces comprise only the time span in which oscillations occur in the impedance signal.

**Table 1:** Cross correlation coefficients.

	cell number *N*	covered area *A*	circularity *<C*>	TIRF intensity	isolated cells *N*_s_
*^ρ^_|Z_*_(*t*)|,y(*t*)_	0.176±0.048	0.138±0.048	0.035±0.02	0.41±0.03	–0.7±0.15

Cross-correlation coefficient *ρ* of the corresponding descriptors, cell number *N*, mean covered area per cell *A*, mean circularity <*C*>, TIRF intensity (time axis multiplied with 1.94 to match periodicity of impedance spikes), and number of isolated cells *N*_s_ with impedance time traces. Amebas were displaying oscillations in impedance recorded at 4 kHz generator frequency.

Only a poor correlation between impedance oscillations and surface coverage with amebas was found as clear oscillations are absent in both fluctuations of number of ameba and mean area. The small correlation might be due to the fact that chemotaxis leads to periodic shape changes or reversible formation of clusters of cells that changes the number of counted amebas on the electrode as well as its mean occupied area.

**Figure 4:**
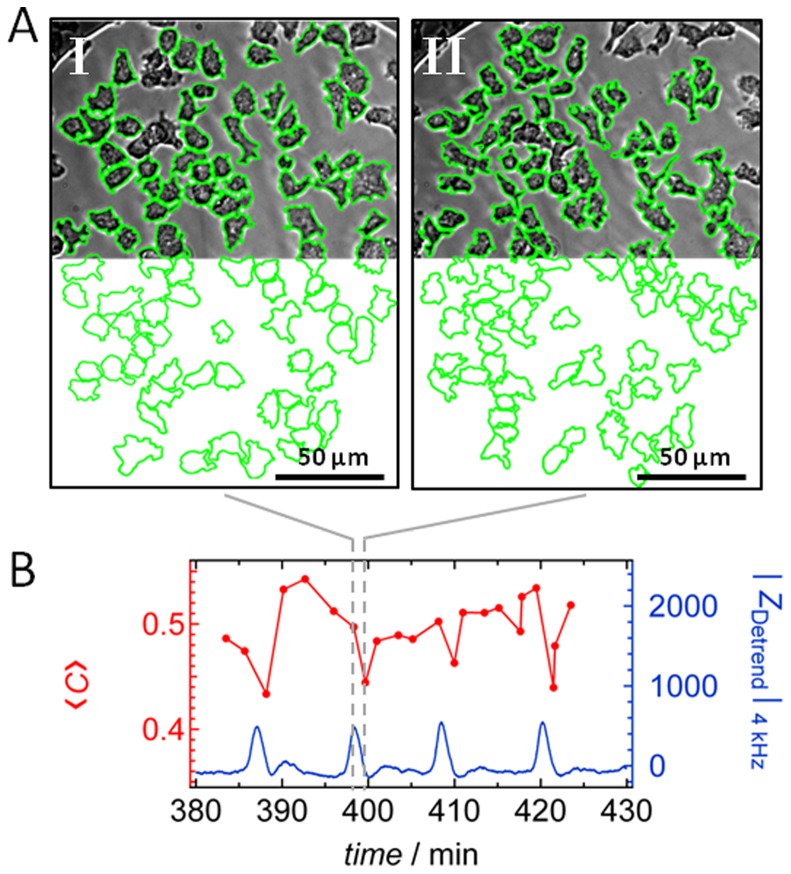
Changes in circularity of *D. discoideum*. A) Optical micrographs of the ameba-covered electrode taken at two different times as marked by the dashed line in (B). Cells’ perimeters were manually surrounded by a green line to compute circularity. For sake of clarity, the electrode is eradicated from the bottom part of the images. B) Circularity <*C*> of *D. discoideum* cells determined as a function of time (red dots). Additionally, the corresponding time series of the detrended impedance values |*Z*_Detrend_|_4 kHz_ from the same area is shown in blue.

Besides cell number and covered area of the electrode, we also related the circularity of cells to the impedance signal as a descriptor for shape changes. The average circularity of an ensemble of cells is defined as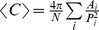, with *N* the overall number of cells, *A_i_* the area of an individual ameba and *P_i_* its perimeter. Cells were again subject to manual segmentation analysis as well as automated cell segmentation as illustrated in Figure 4A to obtain circularity *<C*> as a function of time. Automated segmentation analysis led to quantitatively similar results, however, with less confidence in contour recognition (Fig. S2, Supporting Information). Although circularity was found to oscillate with the same frequency as the impedance signal, the peaks in time traces of the circularity changes were considerably broader compared to the corresponding impedance signal and neither minimal nor maximal circularity corresponds to either maximal or minimal impedance (Fig. 4B). Circularity changes during chemotaxis are also very small in contrast to the expected periodic cringing and elongation of amebas. This is also reflected in a low cross-correlation coefficient of *ρ*_|*Z*(*t*)|,<*C*(*t*)>_ albeit oscillation frequency matches well (Fig. S3 panel D, Supporting Information). Noteworthy, circularity time traces with a very poor signal-to-noise ratio cannot be the origin of the impedance oscillations that display very high signal-to-noise ratio.

### Oscillation of the Cell-substrate Distance

Generally, the distance between cells and substrate largely determines the impedance of adherent cells. Time dependent variations of the cleft between the cell and the electrode give rise to characteristic impedance fluctuations that are usually referred to as micromotion [38]. Micromotion of living cells is highly correlated and often interpreted as fractional Brownian motion following a power law of the power spectral density spectrum with exponents exceeding –2.5 [39]. This highly correlated motion most likely originates from opening and closing of bonds participating in cellular adhesion [37], [40]. Here, we reckon that amebas might change the overall cell to substrate distance in response to cAMP waves giving rise to oscillations that might explain the periodic impedance changes. In order to prove this hypothesis, we devised an experiment that allowed us to correlate the time dependent distance of cells to the substrate measured by TIRF microscopy during the chemotaxis phase qualitatively to the changes in the impedance signal.

**Figure 5:**
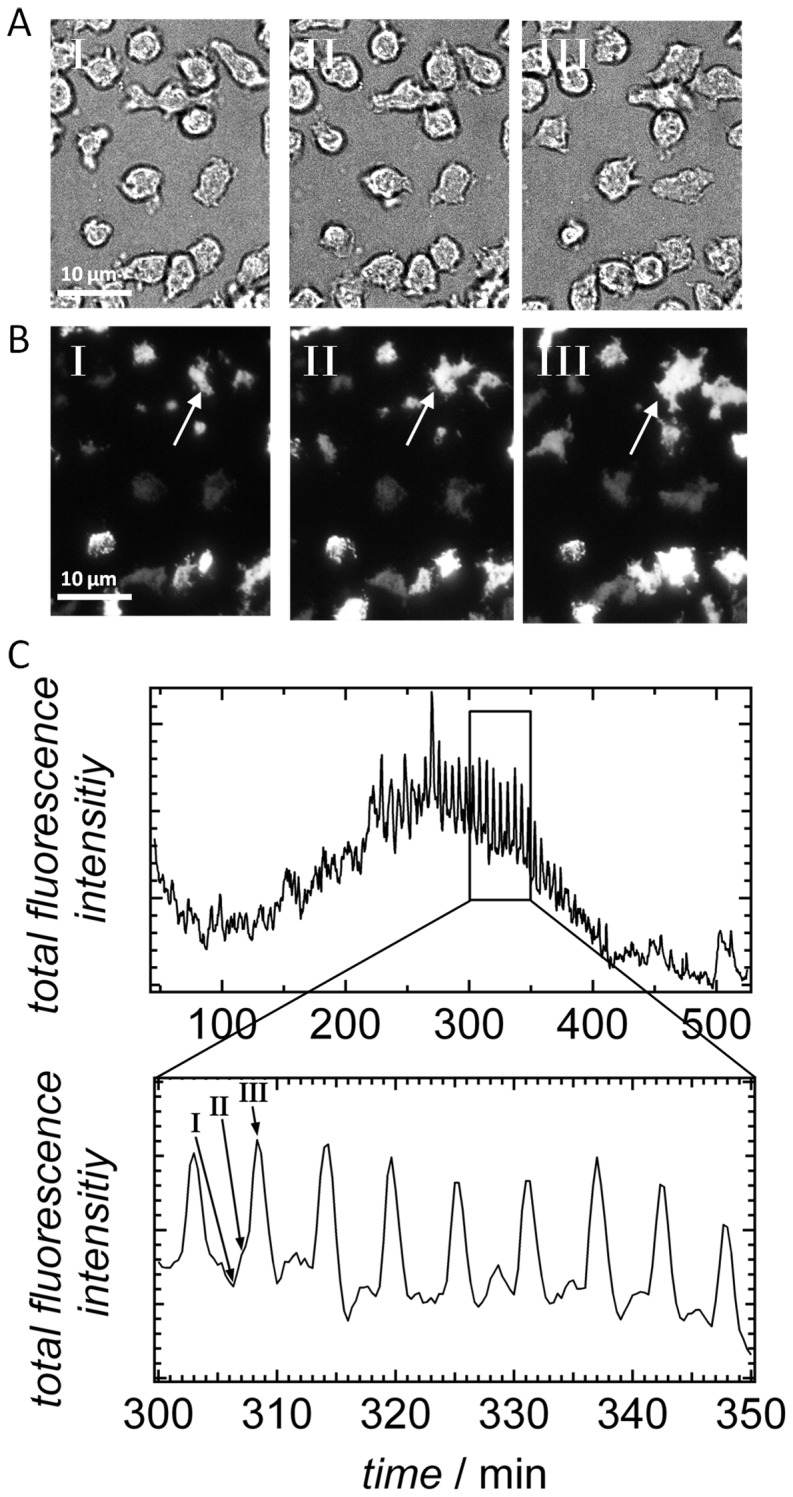
TIRF analysis of *D. discoideum* chemotaxis. A) Bright field and B) TIRF microscopy image sections of cells (8,000 cells mm^-^^2^) starved for 5 h on a glass substrate recorded at three distinct time points (I, II, III). The bright field images are recorded 10 second earlier than the TIRF images presented beneath. The arrow exemplarily highlights one particular cell during the stages I-III. C) Plot of the measured total fluorescence intensity of the whole TIRF image as a function of time shows an oscillation, which is cropped and magnified in the second plot. The first image (I) in B corresponds to a peak minimum of the fluorescence intensity and the third image (III) to a peak maximum.

Figure 5 A/B shows bright field and corresponding fluorescence images of cytosolic GFP-labeled cells, which were starved for 5 hours. The first image of Figure 5B displays only low fluorescence intensity, which is due to a high distance between *D. discoideum* and the substrate surface. On the subsequent images a decrease of the overall cell-substrate distance of cells and their pseudopodia is visible since the fluorescence intensity increases (see Movie S1, Supporting Information). The total fluorescence intensity of each image as a function of time shows periodic signals. Oscillations of the overall TIRF intensity set in 4 h after seeding with a time period of 6 min (Fig. 5C).

**Figure 6:**
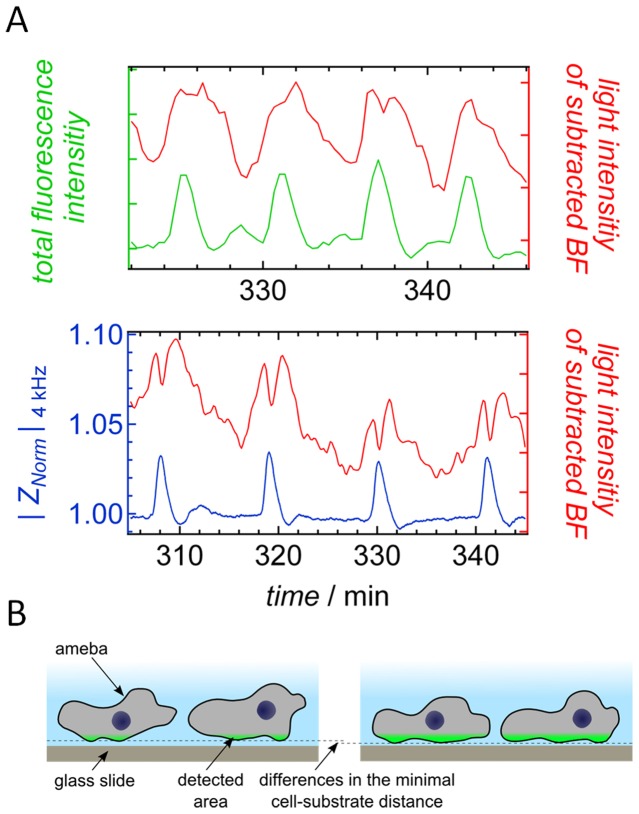
Correlation between TIRF and ECIS experiment. A) Oscillating gray tone of subtracted bright field images (red) in comparison to the total fluorescence intensity of the corresponding TIRF images (green) and the impedance signal (black) as a function of time. The procedure allows to correlate the two independent experiments. B) Scheme of the two proposed states that amebas assume during TIRF microscopy explaining low fluorescence intensity (left) and high intensity (right). The minimal cell-substrate distance is not significantly undercut, only the overall contact zone increases leading to larger fluorescence intensity and impedance.

Since TIRF microscopy cannot be carried out through the substantially opaque ECIS electrodes, we had to find a way to unequivocally correlate the time series of TIRF images with the impedance signal. A time dependent correlation of the impedance signal and the changes in height detected by TIRF microscopy is achieved by comparing and correlating the data with their corresponding bright field images. The procedure works as follows: time elapsed bright field microscopy images of amebas were acquired in two separate experiments, one set of images was acquired on gold electrodes simultaneously with impedance measurements and the other set of images was obtained from glass slides quasi-simultaneously (a corrected time lag of 10 seconds) with TIRF microscopy imaging (see Movie S2 and Fig. S4, Supporting Information). In either case, two bright field images with a time delay of one minute are subtracted providing an overlay image (see Movie S3, Supporting Information). Afterwards, the integral of the magnitude of the overlays is computed and the time dependent fluctuations of the overall gray tone are analyzed (Fig. 6A). We found that these difference images display periodic gray tone fluctuations that could be unequivocally assigned to corresponding oscillations found in fluorescence intensity obtained from TIRF-imaging and oscillations of the impedance signal, respectively (Fig. S5, Supporting Information). Hence, this procedure permits to indirectly relate impedance data with changes in fluorescence intensity due to an overall variation of the cell-substrate distance. We found that high impedance values, the peaks in Figure 2, correlate with the time point of high fluorescence intensity and therefore with a small overall cell-surface distance (Fig. S4–S7, Supporting Information). Since cell densities used for ECIS recordings and TIRF experiments were different the time periods between two maxima (fluorescence intensity or impedance) deviate by roughly a factor of two. Therefore, we corrected for the difference in periodicity by multiplying the time axis with a factor 1.94 corresponding to the difference in time periods between the intensity spikes. As a consequence, we could subject impedance data and TIRF intensities to correlation analysis (Fig. S6–S7, Supporting Information) after rescaling. The correlation was found to be substantial (*ρ*_|*Z*(*t*)|,TIRF(*t*)_ = 0.4) compared to other descriptors such as circularity (Table 1).

In summary, we can conclude that the detected impedance signal of the ECIS experiments is sensitive to, if not dominated, by changes of the cell-surface distance of *D. discoideum* amebas during chemotaxis.

Careful analysis of individual amebas during the chemotaxis shows that each ameba changes between a state in which the cells occupy a larger area in close distance to the surface, being responsible for the overall increased fluorescence intensity, and a state with heavily reduced fluorescence due to a smaller area close to the surface. Remarkably, the maximal fluorescence intensity of the detected area is almost independent of the overall variation of the cell-substrate distance (Fig. 5B). Even if cells display a short overall cell-substrate distance at maximal fluorescence intensity, the minimal distance from the surface is largely preserved, as schematically illustrated in Figure 6B. It is also evident that an increase in fluorescence intensity is correlated with a loss of contrast in the bright field images (see Fig. 5A).

**Figure 7:**
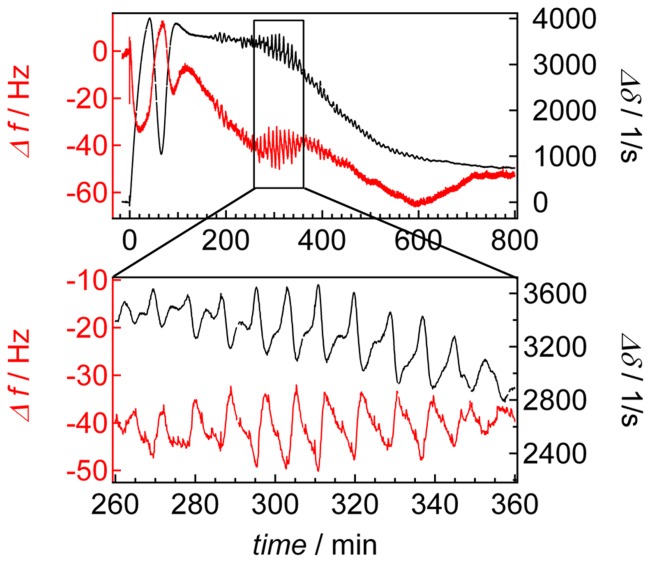
D-QCM of *D. discoideum* chemotaxis. D-QCM measurement of starved *D. discoideum* amebas. Shift in resonance frequency (red) and damping (black) of an oscillating quartz crystal as a function of time. *D. discoideum* cells (10,000 cells mm^−2^) were seeded at *t* = 0 on a gold-electrode. The black box highlights the time period during which collective oscillations occur.

Another way to measure the distance of cells to a surface integrating over a considerably larger area (diameter of the electrode: 3 mm) with extremely high sensitivity is provided by piezoelectric acoustic resonators. The response of thickness shear mode resonator commonly referred to as the quartz crystal microbalance, strongly depends on the coupling of the load to the resonator’s surface, the viscoelastic nature of the foreign mass, its density and dimensions. Cells were seeded on the gold electrode of a quartz resonator oscillating at a fundamental frequency of 5 MHz. The change in resonance frequency and energy dissipation can be used as readout for changes in acoustic load. Figure 7 shows the resonance frequency shift of *D. discoideum* cells seeded on the crystal surface together with temporal changes of its dissipation. The oscillation becomes clearly visible in both readouts 180 min after cell seeding showing a time period of 9 min. Importantly, the peak maxima of the resonance frequency comply with the peak minima of the dissipation and the other way around. This is supported a cross correlation between frequency *f* and dissipation *δ* of *ρ_f_*_(*t*),*δ*(*t*)_ = –0.89. This finding helps to interpret the data in a more detailed manner. In principle, changes in the resonance frequency can be attributed to variations in *mass density*, *electrode coverage*, *viscoelasticity*, *cell-substrate distance*, and to a lesser extent to changes of the *cell height*.

Calculations assuming a three-layer model show that a decrease of cell-substrate distance leads to a shift to lower resonance frequency values concomitant to larger dissipation values [41]. Since the shear waves of the resonator decay to negligible amplitude within the cells (penetration depth <1 µm), changes of cell-height might be excluded as a major modulator of resonance properties. Severe changes of either storage or loss modules that might explain the observed changes in frequency and dissipation are also highly unlikely: only changes of the loss modulus of *D. discoideum* cells on the quartz would display an opposite trend between changes in resonance frequency and dissipation. The model calculations suggest that the detected values of the frequency and dissipation oscillations can mainly be attributed to periodic changes of cell-substrate distance. These findings support that collective oscillations of cell-substrate distance occur during the chemotaxis of *D. discoideum* amebas also on larger length scales (approximate cell numbers on quartz electrode 70000 cells; ECIS electrode 130 cells).

### Collective Oscillations in Cell Density – Periodic Formation of 2-D Clusters

Oscillations of the cell-substrate distance are not the only contribution to the impedance fluctuations generated by amebas. Careful image analysis revealed a second, more subtle and collective process that comprises lateral cell-cell organization. It is known from light scattering experiments in solution that the size of *D. discoideum* aggregates change during a period of the oscillation [34], [42]. Periodic changes of the aggregation might therefore also be conceivable to contribute to the impedance oscillation.

**Figure 8:**
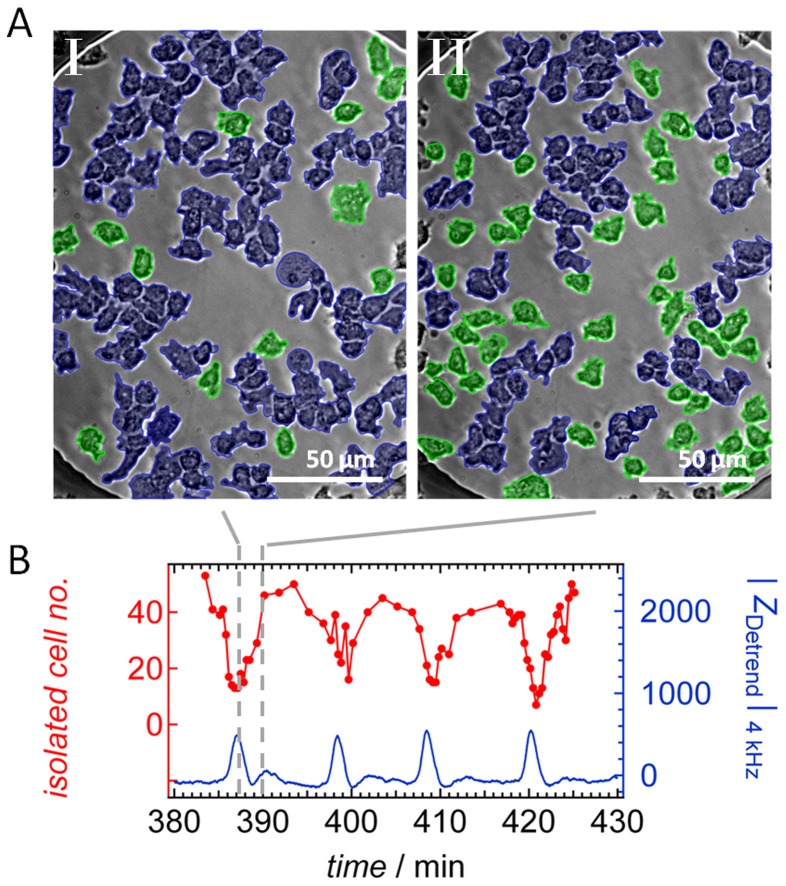
Periodic clustering of *D. discoideum*. A) Optical micrographs (bright field images) of the cell-covered electrode at *t_I_* = 387.2 min and *t*_II_ = 390.2 min after seeding of *D. discoideum* cells (3750 cells mm^−2^). The cells marked in green are isolated amebas, while blue color indicates cells belonging to a 2-D aggregate or cluster. B) Time series of the number of isolated cells derived from image analysis (red curve). Time points labeled with gray lines correspond to the images shown in (A). Additionally, the corresponding time series of the simultaneously acquired detrended impedance values |*Z*_Detrend_| are shown as a blue line.

In order to find correlation between the local arrangement of the *D. discoideum* cells on the electrode and the measured impedance, we manually tracked the amount of isolated cells compared to those associated to clusters. Figure 8A highlights united cell clusters as structures marked in blue color and isolated cells in green. Cells were classified as clustered if cell-cell boundaries are extended over a substantial length scale (conformal contact).

**Figure 9:**
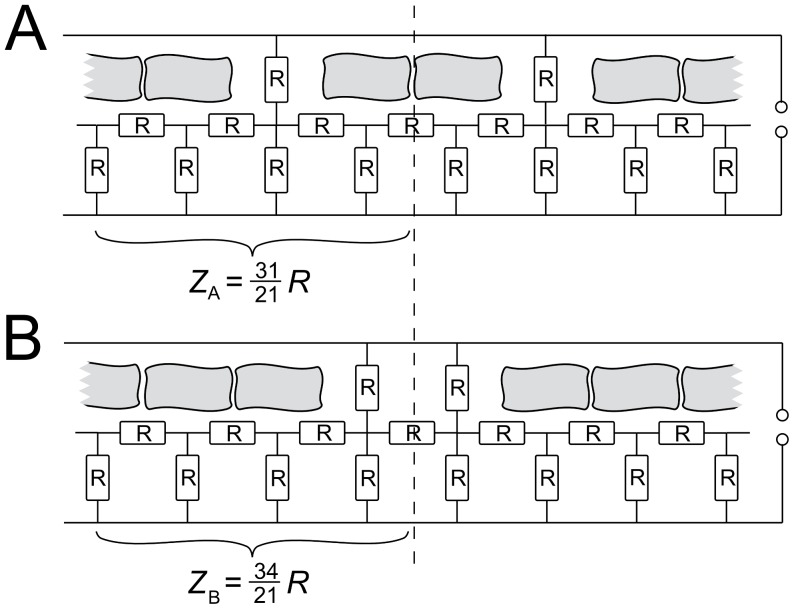
Modeling the impact of clustering on resistance. Illustration of the envisioned impact of distribution of amebas on the electrode. A less-clustered organization (A) with more single amebas produces smaller impedance compared to a more clustered arrangement (B). The impedance depending on the resistant *R* is calculated for one recurring allotment for each case.

By simply counting the number of isolated cells per frame, we obtained the dotted red curve displayed in Figure 8B. Despite the fact that the classification of “clustered” or “isolated” cells is afflicted with a notable error due to limited optical resolution of cell boundaries, the correlation between the number of isolated cells *Ns*(*t*) and impedance is considerable *ρ*_|*Z*(*t*)|,*Ns*(*t*)_ = −0.69 (Table 1). Each peak maximum in the impedance corresponds to a local minimum in isolated cell number. The question was now whether this observation can be cast into a straightforward model explaining why cells organized in a cohesive cluster generate higher impedance values on the electrode than the same number of isolated cells. Modeling the amebas as highly resistive plaques floating in a conductive buffer media, the intercellular spacing plays a distinct role in the total conductivity. Even with a constant surface coverage one can imagine that changes in the spatial distribution of cells are reflected in the overall impedance. A homogeneous distribution of isolated cells results in a homogeneous network of conducting intercellular gaps, while a highly clustered cell distribution exhibits large isolating islands in combination with extended conducting areas. Since the impedance is build up mainly by the electrolyte paths beneath the basal cell membranes, the homogeneous scenario generates lower impedance values while heterogeneities, i.e. due to cluster formation, in the cell distribution lead to rising impedance values. Figure 9 illustrates this effect in a one-dimensional sketch using a simple ohmic model for the overall resistance as a function of arrangement. Here, the surface coverage with isolating cells is kept constant, while the intercellular spacing is changed from a less-clustered organization comprising dimers of cells (9A) to a more clustered situation in which cells are forming trimers (9B). One can easily verify that the resistance in (9A) is lower than in (9B). The resistance ratio between the less-clustered and more clustered state of the one-dimensional case is 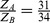.

Therefore local, small scale periodic changes in the lateral arrangements of amebas over time can generate appreciable changes of the overall impedance. Generally, a less clustered organization of cells induces lower impedance values compared to a more clustered arrangement. This process of periodic cell-cell contact formation involves a set of 5–10 participating amebas and must not be mistaken with the periodic large-scale density fluctuations which are observed in the onset of slug formation.

## Discussion

The origin of impedance oscillations of starved *D. discoideum* amebas cultured on micrometer-sized electrodes has been investigated by means of bright-field microscopy, total internal reflection fluorescence microscopy and viscoelastic changes monitored by acoustic resonators. A number of conceivable contributions from changes in shape and number density could be ruled out, while two prominent periodic alterations are identified as the major source for impedance oscillations. On the one hand there is the periodic formation of cell-cell-contacts, i.e. local reversible aggregation of cells into small ensembles that would account for the observed impedance changes. On the other hand - even more substantially - are fluctuations of cell-substrate distances, which also show high correlation with the observed impedance changes. TIRF intensity fluctuations and impedance changes display both a very high signal-to-noise-ratio, an important prerequisite to identify the cause for the observed impedance fluctuations.

**Figure 10:**
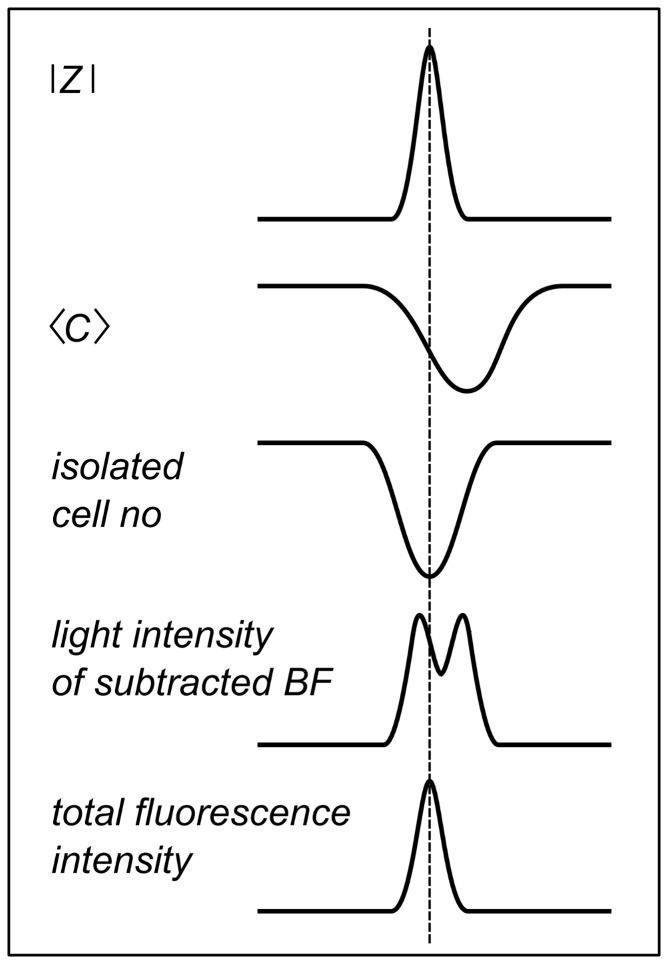
Origin of impedance oscillation . The scheme illustrates how circularity <C>, number of isolated amebas no., light intensity of subtracted bright field BF, and fluorescence intensity from TIRF mages (TIRF) correspond temporally to the measured impedance spikes |*Z*|.

Impedance data were acquired simultaneously to bright-field (BF) images allowing directly correlating number density of amebas, occupied area, shape changes represented by circularity, and fraction of individual to grouped cells with impedance fluctuations. Indirectly, we are able to correlate the cell-substrate distance measured by TIRF microscopy with impedance spikes using simultaneously recorded BF microscopy images (Fig. 6) to establish a cross correlation with subtracted BF-images. We find that the two most obvious reasons for impedance fluctuations, changes in number density and occupied electrode area, only poorly correlate with impedance time traces. Therefore, we conclude that other factors are more important to explain the occurrence of impedance spikes upon starvation (Fig. 10). A commonly accepted change in the general shape of amebas from roundish to an elongated shape occurs during oscillation of cAMP concentration. However, albeit we also find a faint oscillation in circularity that occurs at the same frequency as the impedance fluctuation we could exclude that these shape changes are the main cause of the observed impedance spikes. This is mainly due to two observations. First, the fact that at constant impedance, circularity assumes values over the full range between 0.43 and 0.55 (Fig. 4). Longer time traces evaluated by computer assisted cell segmentation are shown in Supporting Information illustrating this fact with higher resolution. Second, the signal-to-noise ratio of circularity is substantially smaller that that of impedance fluctuations. If circularity was the only cause of impedance fluctuations the signal-to-noise ration should be similar. As a consequence, the cross-correlation coefficient (0.035) is rather low although oscillations of the same frequency are clearly visible (Table 1).

Other sources such as periodic changes in the electrical conductivity and dielectric properties could also be excluded. For instance, Wegener and coworkers could show that changes in membrane capacitance are most prominent at 40 KHz using the same electrode size [37], [43]. At 4 kHz, the frequency we applied in our experiments, the cell substrate distance governs the complex impedance response of the electrochemical system. Measurements with *D. discoideum* carried out at 40 kHz showed less pronounced impedance oscillation than data collected at 4 kHz. This excludes that changes of the capacitance, i.e. changes of the cellular dielectric properties, are responsible for the observed impedance oscillations (data not shown). Additionally, the signal-to-noise ratio of the impedance fluctuation was maximal at 4 kHz. We took this finding as a first indication that the cell-substrate distance plays an important role in these oscillations. Electrical conductivity of the solution influences the impedance spectrum mostly at high frequency [37]. Since the ionic strength of the buffer is several orders of magnitude higher than the change due to periodic cAMP production we exclude changes in buffer concentration as a potential cause of the observed impedance spikes. Therefore, at high frequencies impedance oscillations were not visible.

Electric cell-substrate impedance spectroscopy is extremely sensitive to minuscule changes in the distance of cell from the surface, which is the reason for its name. The same is true for TIRF microscopy. Since simultaneous measurements of TIRF and impedance data are not possible due to the insufficiently transparent gold electrode, which might also quench fluorescence, we had to find a way to correlate both signals in order to find out whether periodic changes of the cell-substrate distance detected in TIRF measurements might be responsible for the spikes in the impedance signal. A closer distance of the cells to the electrode would produce larger impedance since the ionic flux underneath the cells would be restricted to a smaller volume. Correlation between TIRF microscopy and impedance is established by simultaneously using bright field video microscopy in both measurements, which permitted us to relate TIRF-microscopy and impedance time traces as depicted in Figure 4. By subtracting consecutive images that are 1 min apart, it is possible to monitor oscillations in gray level intensity of the images that allowed assigning the time points of high and low impedance to cells close and further away from the electrode. Additionally, the shape of the spikes obtained from TIRF microscopy and those from impedance time traces are substantially similar (*ρ_|Z_*_(*t*)|,TIRF(*t*)_ = 0.4), which is indicative of a conceivable causality between cell-substrate distance and impedance. Considering that the main contributions to impedance changes in ECIS experiments are generally variations in cell-cell distance and cell-substrate distance, a substantial correlation between reduction in cell-substrate distance as observed in TIRF measurements and increased impedance provides a strong case for causality, i.e. the cells form a closer contact to the surface and therefore generate larger impedance. This change in cell-substrate distance occurs in an oscillating manner and has not been described previously.

Acoustic resonator measurements confirm our picture that periodic cell-substrate changes are a major source of impedance oscillations since antidromic changes of resonance frequency and dissipation are a clear sign of changes in the distance of the electrode and the foreign load (amebas).

Changes in cell-substrate distance are, however, not the only determinant of the observed impedance spikes. We found that periodic changes in the aggregation state correlate well with impedance changes. To show that periodic changes of 2D cell aggregation might be responsible for the observed impedance oscillations we devised a simple dielectric model. The model qualitatively explains why clustered cells generate large impedance compared to the same number of isolated cells on the electrode. Chemotaxis of *D. discoideum* comprises both, periodic changes of cell-substrate distance and changes in cell aggregation by forming cell-cell contacts.

### Conclusions

We found that starving *D. discoideum* cells display two kinds of temporal oscillations causing periodic impedance fluctuations of a cell-covered electrode, i.e. changes of the cell-substrate distance *and* reversible formation of small clusters on the electrode surface.

We found that starved amebas periodically reduce their overall distance from the surface producing a larger impedance and higher total fluorescence intensity in TIRF microscopy. This finding was corroborated with D-QCM measurements that show a periodic change in acoustic load assigned to synchronous changes of the cell-electrode distance. TIRF and QCM measurements display a very high signal-to-noise ratio comparable with the S/N ratio of impedance fluctuations. Therefore, we conclude that the dominant source of the observed impedance oscillations is periodic changes of the overall cell-substrate distance of a cell. A decreased cell-substrate distance forces the current to flow along a narrower cleft that inevitably increases the impedance of the electrode. As a consequence, a periodic variation of this distance gives rise to periodic changes in impedance.

Apart from temporal changes in the cell-substrate distance periodic changes of the local 2-D cell-density generate impedance changes in-phase with cell-substrate oscillations, i.e. a lower distance to other adjacent cells corresponds to a higher degree of cluster formation and creates larger impedance under preservation of cell number. Both oscillations in cell-contact formation contribute to the label-free and non-invasive impedance measurements but most likely with different weights. Remarkably, concomitant to periodic changes in cell-substrate and cell-cell contacts, oscillations in circularity, serving as a reporter of cell shape, were also observed, however, without clear correlation to the impedance signal except for the frequency of oscillation. Therefore we propose that *cringing* of amebas, as reported throughout literature, seems to be less significant in this stage of chemotaxis that is rather dominated by periodic changes of the cellular adhesion to the substrate or to neighboring cells. In fact, we did not find rounding up of cells in any stage, which can be correlated to a particular impedance response. General shape changes are rather subtle and do not explain the distinct impedance oscillations. The biological significance of cell-substrate oscillations being in-phase with cell-cell contact formation remains to be elucidated. It is, however, obvious that cells switch between an arrested state, in which they also form clusters and adhere more strongly to the surface, and a highly mobile state where single ameba migrate with only minor attachment to the surface.

## Experimental Methods

### Cell Culture

The AX3 WT strain and the cytosolic expressing GFP cell line AX2 HG1694 of *D. discoideum* (kindly provided by the Gerisch lab [44]) are used as indicated. In HG1694 cells, GFP is overexpressed under the control of an Act15 promotor. The HG1694 strain produces GFP that is not conjugated to another protein, which makes it freely diffusible in the cytosol. Variations in fluorescence intensity from cell to cell might be attributed to a different GFP expression level. The cells were cultivated in HL-5 medium with glucose from ForMedium (Norfolk, UK) at 22°C on polystyrene dishes (Primaria, Falcon) and renewed from frozen stock every four weeks. For HG1694 cultivation the addition of antibiotic was necessary (0.05 wt % Geneticin (G418), Roche, Basel, Gibco).

For ECIS, D-QCM, microscopy, and TIRF measurements, cells were collected by centrifugation, washed twice and were resuspended in Sorensen’s buffer (SB; 14.6 mM KH_2_PO_4_, 2 mM Na_2_HPO_4_, pH 6.1) (4 × 10^6^ cells per mL).

### Electric Cell–Substrate Impedance Sensing (ECIS)

Time-resolved impedance data was acquired with a home-made setup [40] consisting of an impedance analyzer (Si 1260, Solartron, Hampshire, UK) and a PC for experiment control and data storage. In our setup, a 30 mV AC signal is applied to the system and the complex impedance recorded at 4 kHz with a sampling rate of 0.7 Hz. *D. discoideum* cells (3750 cells mm^−2^) were added to 8W1E ECIS arrays (Applied Biophysics, Troy, NY), where each well contains a small circular gold electrode (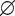 = 250 µm) and a very large counter electrode with an area of 0.15 cm^2^ to limit impedance response to the smaller working electrode. Impedance oscillations are observed in over 50 independent experiments. The gold film was slightly transparent, which allowed to perform optical inspection using an inverted light microscope. For reducing long-term trends the time series data was detrended by a moving average algorithm with a box size of 800 points.

### Microscopy

Bright field (BF) microscopy in combination with ECIS-experiments was carried out with an inverted microscope (IX81 Olympus, Tokyo, Japan) controlled by the Olympus software xCellenceRT. Images of *D. discoideum* amebas were acquired with a CCD camera (XM10, Olympus, Tokyo, Japan) using a LUCPLFLN 40x lens (Olympus, Tokyo, Japan) at a sampling rate of 10 frames per second.

### Total Internal Reflection Fluorescence (TIRF)-microscopy

BF and TIRF microscopy have been carried out on the same Olympus IX81 (Olympus, Tokyo, Japan) setup. Images of *D. discoideum* were obtained with a Plan Apo N 60x oil immersion objective (Olympus, Tokyo, Japan). The cytosolic GFP expressing cell line HG1694 was plated at a density of 4–5×10^6^ ml^−1^ on a glass bottomed Petri dish (No. 1.5, diameter 50 mm, MaTek Corporation, Ashland, MA) and starved in SB. Cells were excited by the 488 nm line of a 20 mW Ar laser. BF and TIRF images were acquired with a 16 bit CCD camera at 1344×1024 pixels (Hamamatsu Photonics, Hamamatsu, Japan). Experiments were carried out in triplicate.

### Image Analysis and Cell Segmentation

For analysis of optical micrographs, the contours of the cells were either manually surrounded in the software MyPaint (freeware) on a graphic tablet (Wacom Europe, Krefeld, Germany) or automatically detected with self-written software. The following image evaluations were carried out with Matlab (The MathWorks, Notick, MA). We assign cells to clusters if a common cell boundary between two or more cells was found by segmentation analysis. Otherwise, cells were counted as individual, single cells.

### Quartz Crystal Microbalance in Dissipation Mode (D-QCM)

Changes in resonance frequency and dissipation were measured with a home-made D-QCM setup as described previously [45], [46] equipped with 5 MHz AT-cut quartz crystals (KVG, Neckarbischofsheim, Germany) with circular gold electrodes. Amebas are seeded at a density of 8×10^5^ cells per mL (10,000 cells mm^−2^) directly onto QCM quartzes. Experiments were carried out in triplicate.

## Supporting Information

Figure S1
**Normalized impedance |** ***Z_Norm_*** **|_4 kHz_ of** ***D. discoideum*** **amebas in glucose-free buffer on an ECIS electrode (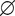 = 250 µm).**
(PNG)

Figure S2 **Detrended time traces, i.e. fluctuations of cell number Δ** ***N*** **, covered area Δ** ***A*** **, circularity Δ** ***C*** **with corresponding impedance data Δ** ***Z*** **(blue) computed from automated cell segmentation analysis of bright field images.**
(PNG)

Figure S3 **A) Time interval between two maxima of impedance peaks (blue triangles) and corresponding intensity maxima from integrated bright field subtraction images (red circles) obtained from the electrode as a function of period number.** B) Time interval between two maxima of TIRF (total internal reflection fluorescence) intensity peaks (green triangles) and their corresponding intensity maxima from bright field subtraction images (red circles) as a function of period number. C) Time interval between two subsequent maxima of impedance spikes (blue circles), two corresponding maxima of the number of single amebas (green triangles), and two corresponding maxima of circularity (red circles) as a function of period number. D) Time interval between two impedance maxima (blue circles) and two corresponding spikes of circularity (red circles) evaluated by an automated cell segmentation software.
(PNG)

Figure S4 **A) Detrended ECIS-oscillations (blue) and corresponding bright-field subtraction intensities.** B) Detrended TIRF-oscillations (blue) with corresponding bright-field subtraction intensities.
(PNG)

Figure S5 **A) Autocorrelation functions of detrended impedance data, TIRF intensity and corresponding bright field subtraction intensities of oscillating** ***D. discoideum*** **amebas.** The data shows the periodicity of the signal and confirms that intensities from BF subtraction images exhibit identical periodicity as the corresponding ECIS and TIRF experiments, respectively. B) Cross-correlation of BF subtraction images with impedance recording (ECIS, blue) and TIRF intensities (red).
(PNG)

Figure S6 **Cross correlation-coefficient (corr.coef.) of ECIS and TIRF data as a function of time stretching factor for TIRF-intensities.** The maximal correlation is found for a TIRF stretching factor matching the two periods (app. 1.9–2) in the time domain. The high correlation-coefficients implies that the peak shapes of the two spikes from ECIS and TIRF measurements during chemotaxis of *D. discoideum* amebas are very similar.
(PNG)

Figure S7 **Cross-correlation of ECIS and TIRF signals.** The time axis of the TIRF signal was multiplied with 1.94 (the maximum correlation obtained from figure S6) in order to remove the differences in oscillation period due to cell density differences. Cross-correlation is substantial and persistent during cAMP oscillations.
(PNG)

Movie S1 **TIRF microscopy video of** ***D. discoideum*** **cells (8000 cells/mm^2^) starved for 5 h on a glass substrate representing 40 min taken at 6 fps.**
(AVI)

Movies S2 **Bright field microscopy video of** ***D. discoideum*** **cells (8000 cells/mm^2^) starved for 5 h on a glass substrate representing 40 min taken at 6 fps corresponding to Movie S1.**
(AVI)

Movies S3 **Subtracted bright field microscopy video of** ***D. discoideum*** **cells (8000 cells/mm^2^) starved for 5 h on a glass substrate representing 40 min taken at 6 fps corresponding to Movie S2 (see experimental methods).**
(AVI)
