# Supplementary figures and images for: Chemotaxis of Dictyostelium discoideum: Collective Oscillation of Cellular Contacts
Source: PLoS One. 2013 Jan 17;8(1):e54172. doi: 10.1371/journal.pone.0054172 (PMC3547869; doi:10.1371/journal.pone.0054172)

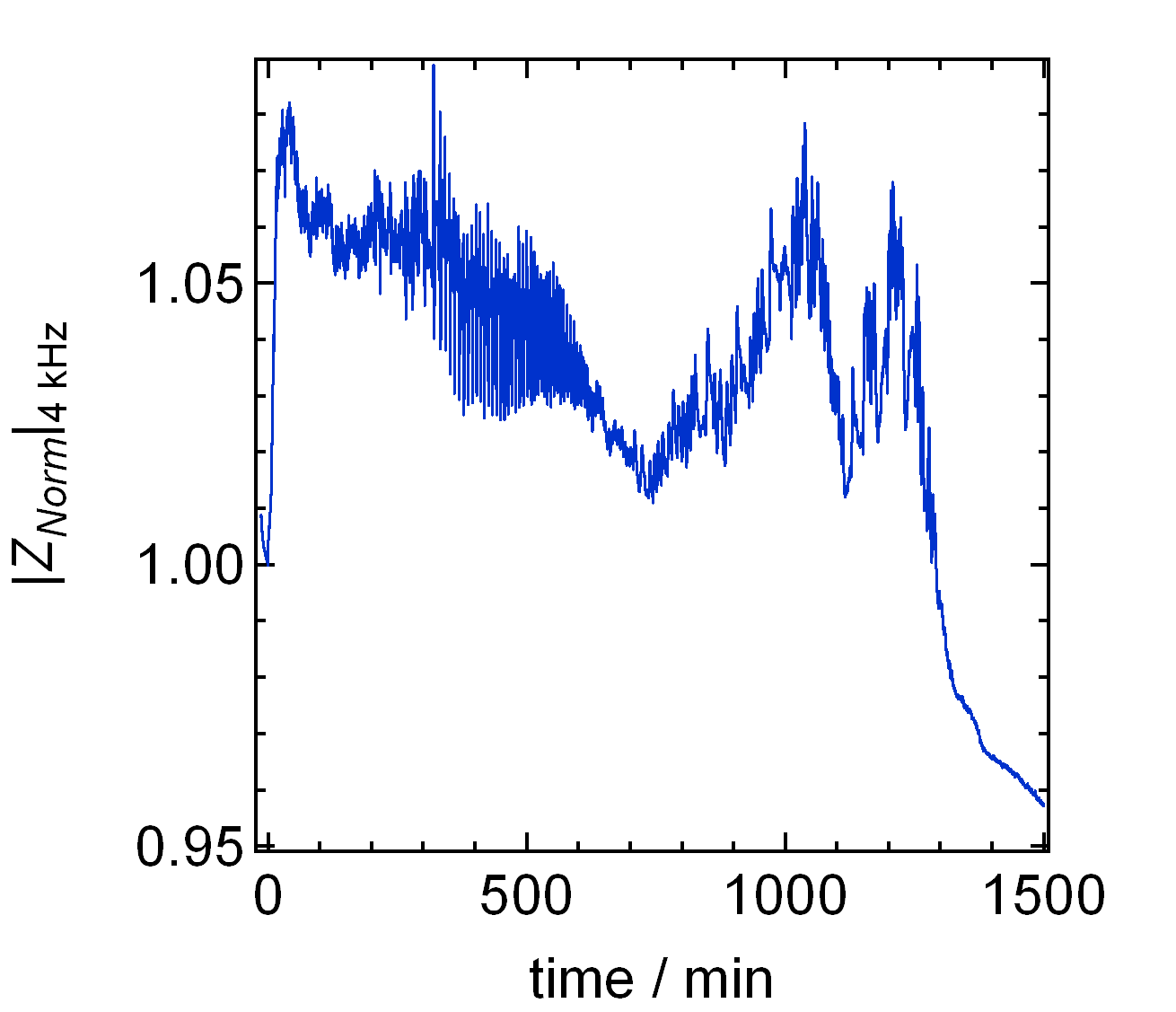

Supplement: Figure S1 — Normalized impedance | ZNorm |4 kHz of D. discoideum amebas in glucose-free buffer on an ECIS electrode ( = 250 µm). (PNG) [file pone.0054172.s001.png]

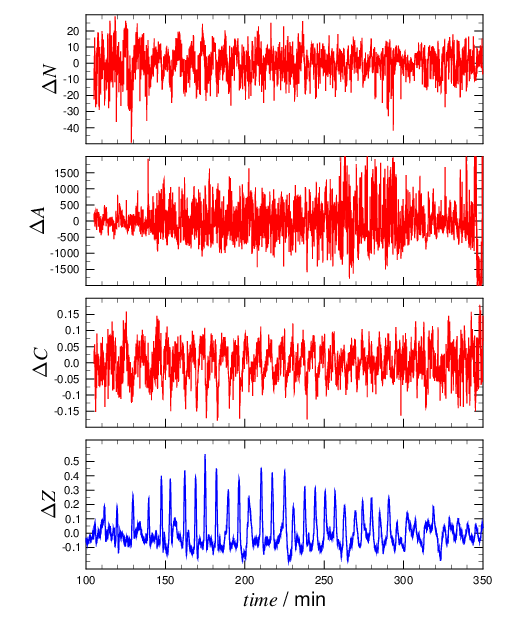

Supplement: Figure S2 — Detrended time traces, i.e. fluctuations of cell number Δ N , covered area Δ A , circularity Δ C with corresponding impedance data Δ Z (blue) computed from automated cell segmentation analysis of bright field images. (PNG) [file pone.0054172.s002.png]

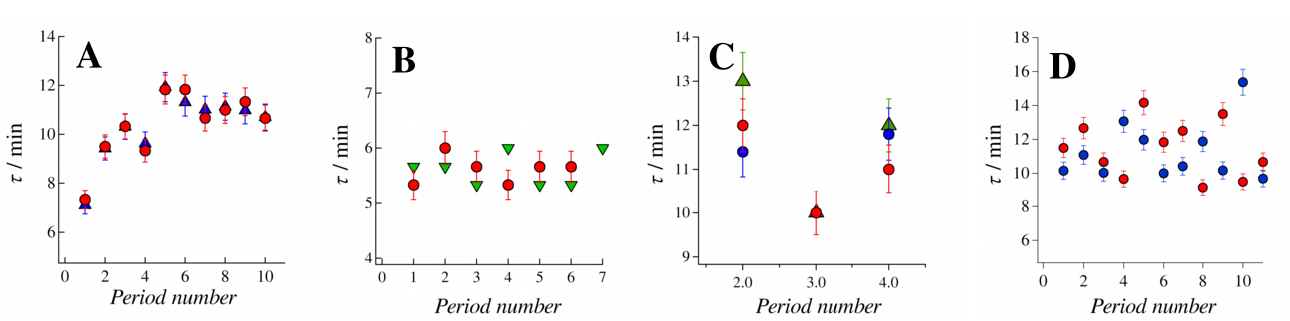

Supplement: Figure S3 — A) Time interval between two maxima of impedance peaks (blue triangles) and corresponding intensity maxima from integrated bright field subtraction images (red circles) obtained from the electrode as a function of period number. B) Time interval between two maxima of TIRF (total internal reflection fluorescence) intensity peaks (green triangles) and their corresponding intensity maxima from bright field subtraction images (red circles) as a function of period number. C) Time interval between two subsequent maxima of impedance spikes (blue circles), two corresponding maxima of the number of single amebas (green triangles), and two corresponding maxima of circularity (red circles) as a function of period number. D) Time interval between two impedance maxima (blue circles) and two corresponding spikes of circularity (red circles) evaluated by an automated cell segmentation software. (PNG) [file pone.0054172.s003.png]

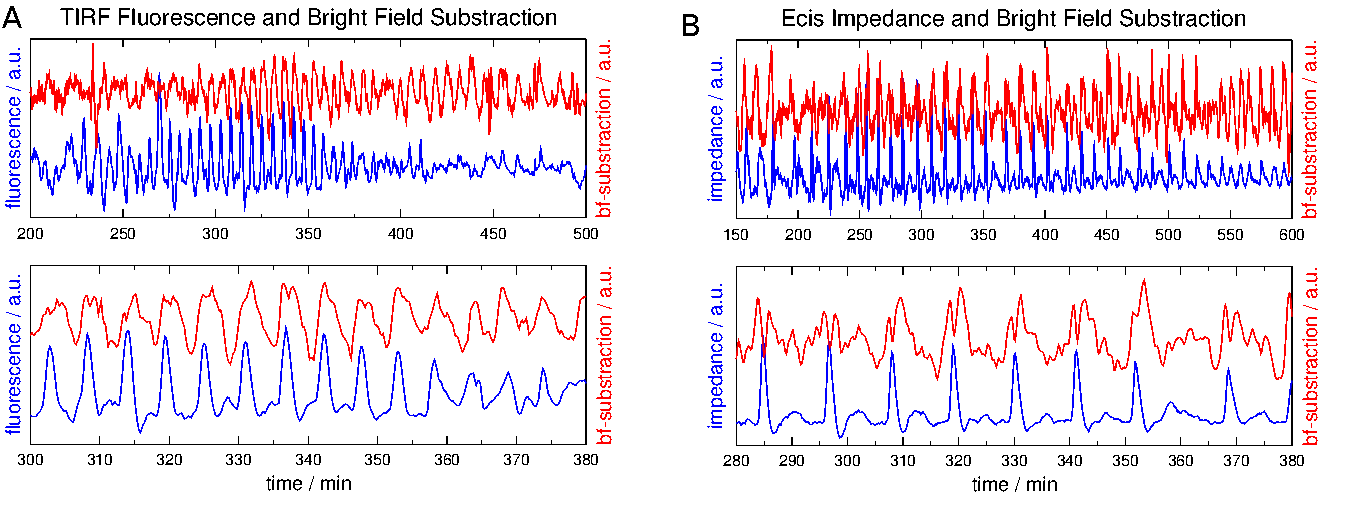

Supplement: Figure S4 — A) Detrended ECIS-oscillations (blue) and corresponding bright-field subtraction intensities. B) Detrended TIRF-oscillations (blue) with corresponding bright-field subtraction intensities. (PNG) [file pone.0054172.s004.png]

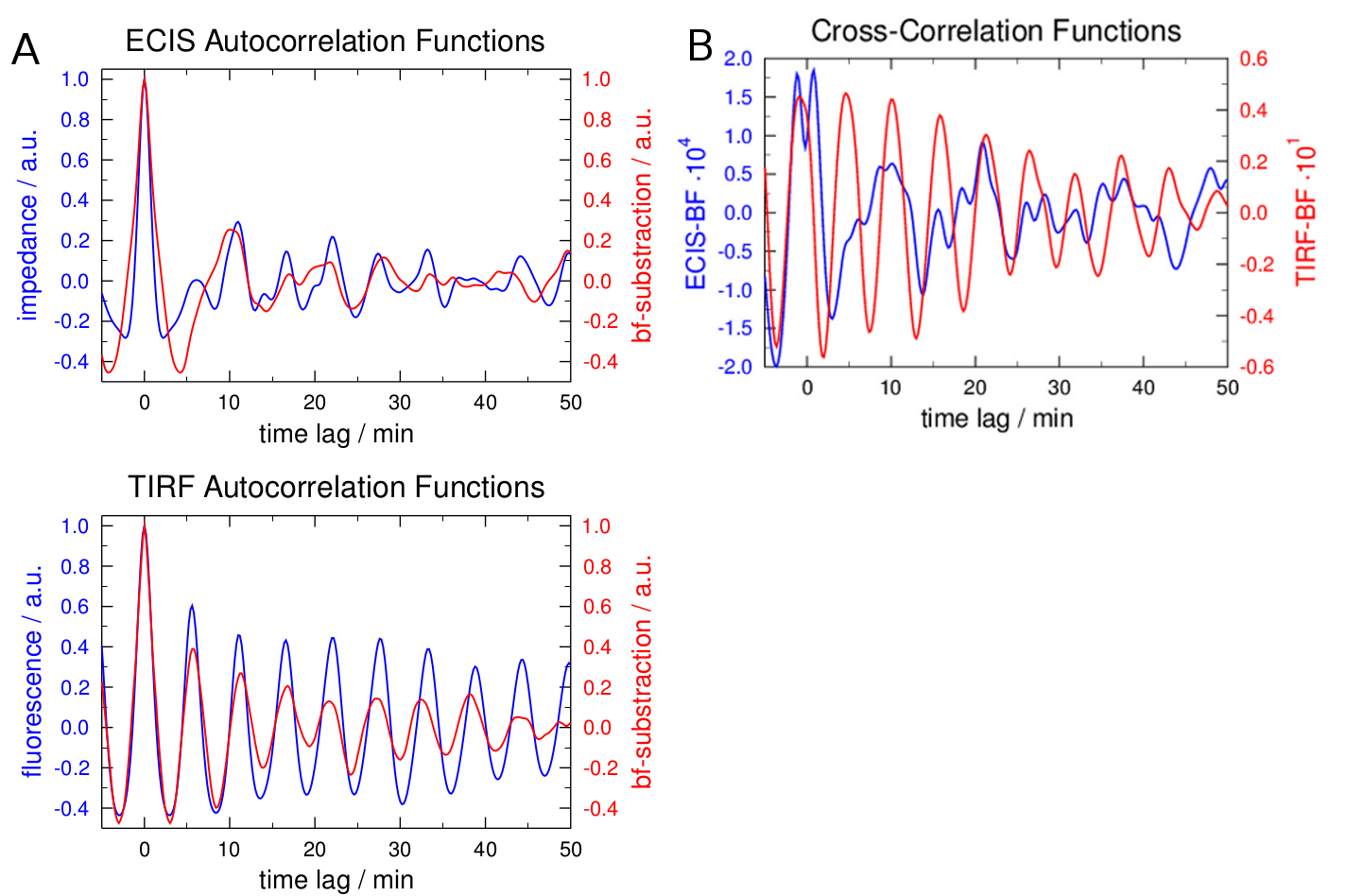

Supplement: Figure S5 — A) Autocorrelation functions of detrended impedance data, TIRF intensity and corresponding bright field subtraction intensities of oscillating D. discoideum amebas. The data shows the periodicity of the signal and confirms that intensities from BF subtraction images exhibit identical periodicity as the corresponding ECIS and TIRF experiments, respectively. B) Cross-correlation of BF subtraction images with impedance recording (ECIS, blue) and TIRF intensities (red). (PNG) [file pone.0054172.s005.png]

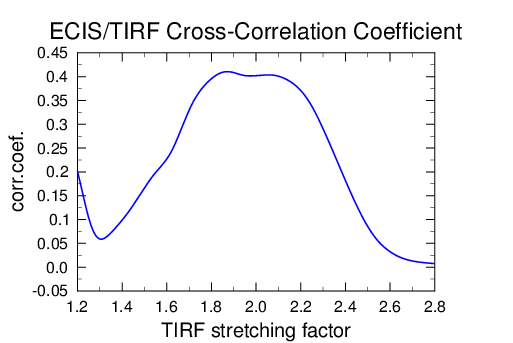

Supplement: Figure S6 — Cross correlation-coefficient (corr.coef.) of ECIS and TIRF data as a function of time stretching factor for TIRF-intensities. The maximal correlation is found for a TIRF stretching factor matching the two periods (app. 1.9–2) in the time domain. The high correlation-coefficients implies that the peak shapes of the two spikes from ECIS and TIRF measurements during chemotaxis of D. discoideum amebas are very similar. (PNG) [file pone.0054172.s006.png]

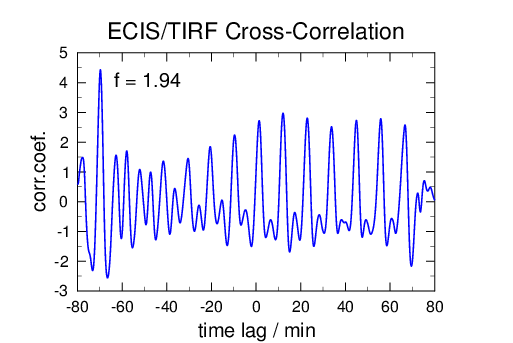

Supplement: Figure S7 — Cross-correlation of ECIS and TIRF signals. The time axis of the TIRF signal was multiplied with 1.94 (the maximum correlation obtained from figure S6) in order to remove the differences in oscillation period due to cell density differences. Cross-correlation is substantial and persistent during cAMP oscillations. (PNG) [file pone.0054172.s007.png]
